# TPM2 as a potential predictive biomarker for atherosclerosis

**DOI:** 10.18632/aging.102231

**Published:** 2019-09-05

**Authors:** Ling-Bing Meng, Meng-Jie Shan, Yong Qiu, Ruomei Qi, Ze-Mou Yu, Peng Guo, Chen-Yi Di, Tao Gong

**Affiliations:** 1 Neurology Department, Beijing Hospital, National Center of Gerontology, Beijing 100730, P. R. China; 2 Graduate School, Chinese Academy of Medical Sciences and Peking Union Medical College, Beijing 100730, P. R. China; 3 Anesthesiology Department, Beijing Hospital, National Center of Gerontology, Beijing 100730, P. R. China; 4 The MOH Key Laboratory of Geriatrics, Beijing Hospital, National Center of Gerontology, Beijing 100730, P. R. China; 5 Department of Neurology, Peking University First Hospital, Beijing 100034, P. R. China; 6 Department of Orthopedics, The Fourth Hospital of Hebei Medical University, Shijiazhuang Development Zone, Hebei 050011, P.R. China; 7 School of Basic Medicine, Peking University, Beijing 100191, P. R. China

**Keywords:** cardiac-cerebral vascular diseases, atherosclerosis, differentially expressed genes, protein-protein interaction, bioinformatics analysis

## Abstract

Cardiac-cerebral vascular disease (CCVD), is primarily induced by atherosclerosis, and is a leading cause of mortality. Numerous studies have investigated and attempted to clarify the molecular mechanisms of atherosclerosis; however, its pathogenesis has yet to be completely elucidated. Two expression profiling datasets, GSE43292 and GSE57691, were obtained from the Gene Expression Omnibus (GEO) database. The present study then identified the differentially expressed genes (DEGs), and functional annotation of the DEGs was performed. Finally, an atherosclerosis animal model and neural network prediction model was constructed to verify the relationship between hub gene and atherosclerosis. The results identified a total of 234 DEGs between the normal and atherosclerosis samples. The DEGs were mainly enriched in actin filament, actin binding, smooth muscle cells, and cytokine-cytokine receptor interactions. A total of 13 genes were identified as hub genes. Following verification of animal model, the common DEG, Tropomyosin 2 (TPM2), was found, which were displayed at lower levels in the atherosclerosis models and samples. In summary, DEGs identified in the present study may assist clinicians in understanding the pathogenesis governing the occurrence and development of atherosclerosis, and TPM2 exhibits potential as a promising diagnostic and therapeutic biomarker for atherosclerosis.

## INTRODUCTION

Cardiac-cerebral vascular diseases (CCVDs) have a high prevalence, disability and mortality rates, and are a serious threat to human health, especially to the health of the populace aged over 50 years [1]. Each year, 15 million people die from CCVDs, which are primarily induced by atherosclerosis, and are the leading cause of mortality in the world [2]. Numerous studies [3–7] have demonstrated that the pathophysiological process for the development of atherosclerosis are closely associated with the mutation and abnormal expression of genes, which include fms-like tyrosine kinase-1 (Flt-1), tumor necrosis factor (TNF)-α, apolipoprotein A-I (apoA-I), vascular endothelial growth factor (VEGF) and angiogenin. A previous study demonstrated that the low-expression of Flt-1 may predict the occurrence of endothelial injury, which subsequently results in the occurrence of atherosclerosis [3]. The stronger the proliferation competence of endothelial progenitor cell (EPC) is, the less feeble endothelial injury may be, and the overexpression of TNF-α may damage the process of EPC development [4]. In addition, the mutation of Apolipoprotein A-I (apoA-I), an anti-atherogenic gene, has been hypothesized to accelerate the apoptosis of vascular endothelial cells and downregulate the levels of eNOS and heme oxygenase-1, which culminates in atherosclerosis plaque formation [5]. It is well established that the expression of VEGF and ANG stimulates the renewal of vascular endothelial cells. Therefore, abnormal inactivation of VEGF and angiogenin expression may exert a pivotal function in the occurrence and development of atherosclerosis [6, 7]. However, due to the lack of timely detection, dynamic monitoring and effective control of the occurrence and progression of atherosclerotic stenosis and vulnerable plaques, the occurrence and recurrence of ischemic CCVDs still cannot be effectively controlled, hence why research investigating ischemic cardiac-cerebral vascular diseases is one of the principal areas of research at home and abroad [8]. Therefore, it is imperative to explore the accurate molecular targets included in the hyperplasia and recidivation of atherosclerosis, in order to make a contribution to the diagnosis and treatment of atherosclerotic diseases.

Since the 21^st^ century, bioinformatics technology has been increasingly used to excavate the potential genetic targets of diseases, which has assisted researchers in authenticating the differentially expressed genes (DEGs) and underlying pathways that are associated with the occurrence and recurrence of atherosclerosis [9–12].

However, it is difficult to acquire credible results when using the independent microarray technology owing to the higher false-positive rates [13]. Therefore, the present study downloaded and analyzed two human expression profiling datasets from the Gene Expression Omnibus (GEO) Dataset, and identified the DEGs between non-atherosclerotic tissues and atherosclerotic tissues. The molecular mechanisms of the occurrence and development of atherosclerosis were subsequently explored via enrichment analysis of functions and pathways, protein-protein interaction (PPI) network analyses and co-expression network analyses, and a total of 234 DEGs and 13 hub genes were identified and authenticated.

In addition, in order to verify the results of the bioinformatics analysis, an animal experiment using two groups (a control and atherosclerosis model group) was implemented. The data of animal experiment was digged via a proteomics assay, and differentially expressed proteins were identified between the control and atherosclerosis groups. Then after comparing the bioinformatics result and proteomic data, Tropomyosin 2 (TPM2) was identified as a commonly differentially expressed gene, which exhibits potential as a molecular target or biomarker for atherosclerosis. A series of low flux experiments were subsequently performed to verify the role and function of TPM2 in atherosclerosis.

## RESULTS

### Validation of the datasets

**Figure 1:**
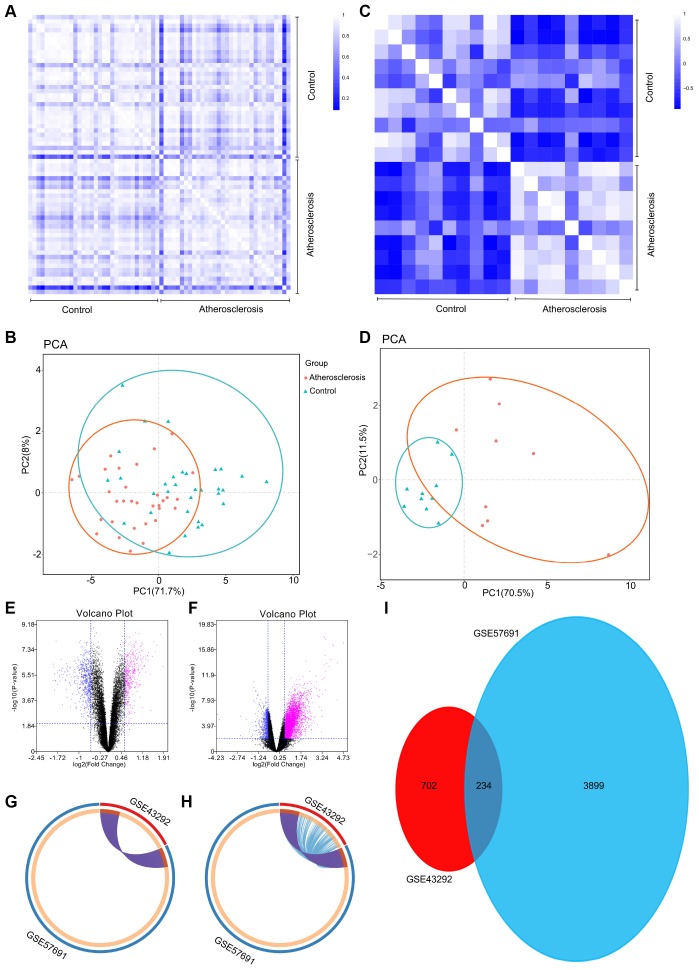
(**A**) Pearson’s correlation analysis of samples from the GSE43292 dataset. The color reflects the intensity of the correlation. When 0<correlation<1, there exists a positive correlation. When -1<correlation<0, there exists a negative correlation. The larger the absolute value of a number the stronger the correlation. (**B**) PCA of samples from the GSE43292 dataset. In the figure, principal component 1 (PC1) and principal component 2 (PC2) are used as the X-axis and Y-axis, respectively, to draw the scatter diagram, where each point represents a sample. In such a PCA diagram, the farther the two samples are from each other, the greater the difference is between the two samples in gene expression patterns. (**C**) Pearson’s correlation analysis of samples from the GSE57691 dataset. The color reflects the intensity of the correlation. (**D**) PCA of samples from the GSE57691 dataset. (**E**) The volcano plot illustrates the differences between control and atherosclerotic tissues after analysis of the GSE43292 dataset with GEO2R. (**F**) The volcano plot illustrates the difference between control and atherosclerotic tissues after analysis of the GSE57691 dataset with GEO2R. (**G**) Overlap between differently expressed gene lists of GSE43292 and GSE57691 only at the gene level, where purple curves link identical genes; (**H**) Overlap between differently expressed gene lists of GSE43292 and GSE57691 not only at the gene level, but also at the shared term level, where blue curves link genes that belong to the same enriched ontology term. The inner circle represents gene lists, where hits are arranged along the arc. Genes that hit multiple lists are colored in dark orange, and genes unique to a list are shown in light orange. (**I**) The Venn diagram could demonstrate that 234 genes were contained in the GSE43292 and GSE57691 datasets simultaneously.

To further validate the intra-group data repeatability, we employed the Pearson’s correlation test and principal component analysis (PCA). Based on the Pearson’s correlation test, we found that in the GSE43292 dataset there were strong correlations among the samples in the control group and that there were also strong correlations among the samples in the atherosclerosis group (Figure 1A). Based on the PCA the intra-group data repeatability for GSE43292 was acceptable. The distances between per samples in the control group were close and the distances between per samples in the atherosclerosis group were also close in the dimension of principal component-1 (PC1) (Figure 1B). Based on Pearson’s correlation test, we found that for GSE57691 there was a strong correlation among the samples in the control group and a strong correlation among the samples in the atherosclerosis group (Figure 1C). The PCA showed the intra-group data repeatability to be acceptable in the GSE57691 dataset. The distances between per samples in the control group were close, and distances between per samples in the atherosclerosis group were also close in the dimension of PC1 (Figure 1D).

### DEGs identified between control and atherosclerotic tissues

Following the analysis of the GSE43292 and GSE57691 datasets with GEO2R, the differences between control and atherosclerotic tissues were presented in volcano plots (Figure 1E and 1F). The Circos present the overlap between differently expressed gene lists of GSE43292 and GSE57691 not only at the gene level (Figure 1G), but also at the shared term level (Figure 1H). These results were standardized, and a total of 936 DEGs in the GSE43292 dataset, and 4,133 DEGs in the GSE57691 dataset were identified. A Venn diagram was constructed, and revealed that 234 genes were simultaneously contained within the 2 examined datasets (Figure 1I).

### Construction of co-expression modules by weighted gene co-expression network analysis (WGCNA)

**Figure 2:**
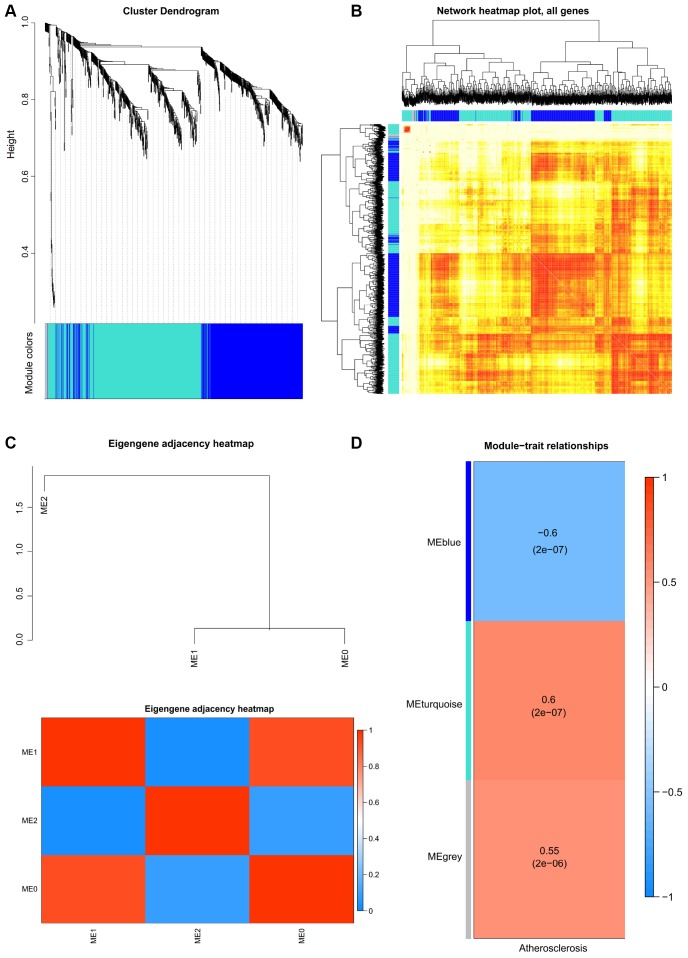
(**A**) Construction of co-expression modules by weighted gene co-expression network analysis (WGCNA) package in R. The cluster dendrogram of genes in GSE43292. Each branch in the figure represents one gene, and every color below represents one co-expression module. (**B**) Interaction relationship analysis of co-expression genes. Different colors of horizontal axis and vertical axis represent different modules. The brightness of yellow in the middle represents the degree of connectivity of different modules. There was no significant difference in interactions among different modules, indicating a high-scale independence degree among these modules. (**C**) Heatmap plot of the adjacencies in the hub gene network. (**D**) Heatmap of the correlation between module eigengenes and the disease status of atherosclerosis. The turquoise module was the most positively correlated with status, and the blue module was the most negatively correlated with status.

One key step is selection of the soft-thresholding power in the procedure of performing a WGCNA. The network topology analysis was performed for thresholding powers and identified the relatively balanced scale independence and mean connectivity. The power value 9, which was the lowest power for the scale-free topology fit index on 0.9, was selected to produce a hierarchical clustering tree (dendrogram) of 936 genes. We set the MEDissThres as 0.25 to merge similar modules, and 3 important modules were generated (Figure 2A). The network heatmap plot shows that there was no significant difference in interactions among different modules, indicating a high-scale independence degree among these modules (Figure 2B). Similar results were demonstrated by the eigengene adjacency heatmap (Figure 2C). The turquoise module was the most positively correlated with status of atherosclerosis, and the blue module was the most negatively correlated with status of atherosclerosis (Figure 2D).

### Functional annotation of DEGs by GO and KEGG analyses

**Figure 3:**
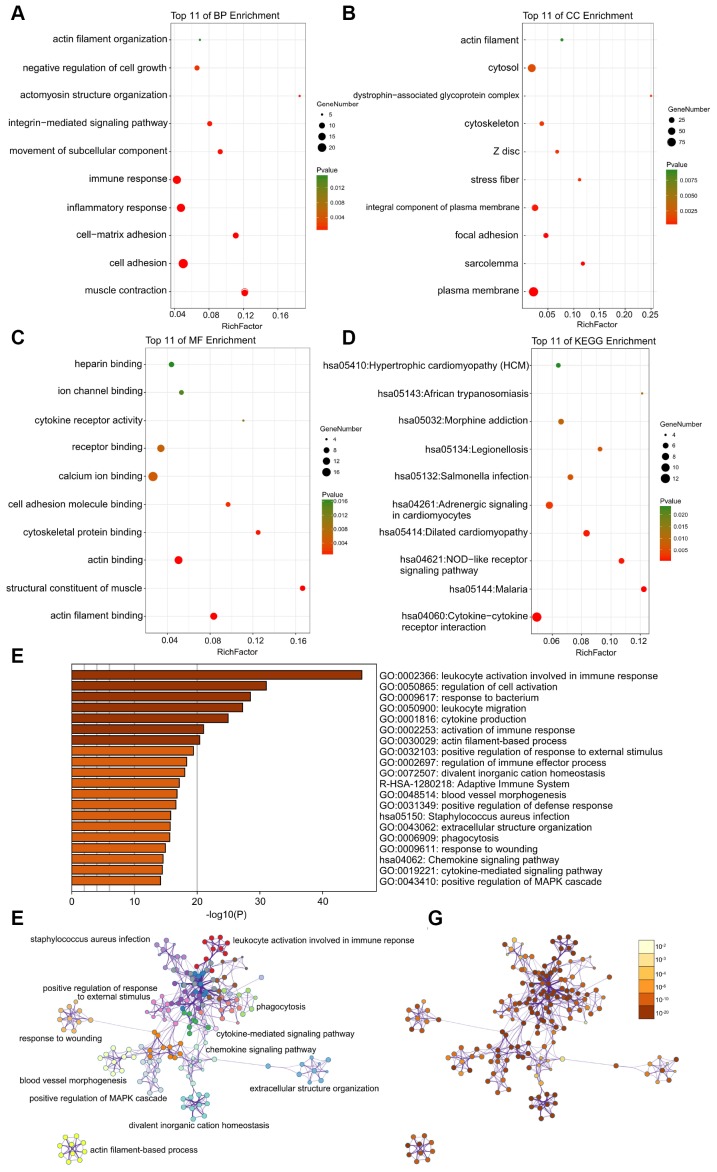
(**A**–**C**) Detailed information relating to changes in the biological processes (BP), cellular components (CC), and molecular functions (MF) of DEGs in atherosclerosis and control tissues through the GO enrichment analyses. (**D**) The KEGG pathway analysis of DEGs. KEGG, Kyoto Encyclopedia of Genes and Genomes; GO, Gene Ontology; DEGs, differentially expressed genes. (**E**) Heatmap of enriched terms across input differently expressed gene lists, colored by p-values, via the Metascape. (**F**) Network of enriched terms colored by cluster identity, where nodes that share the same cluster identity are typically close to each other. (**G**) Network of enriched terms colored by p-value, where terms containing more genes tend to have a more significant p-value.

The DAVID results of the GO analysis revealed that there were main variations in biological processes (BP), including muscle contraction, actin filament organization, and so on (Figure 3A). The variations in cell components (CC) of DEGs were markedly enriched in the actin filament, and cytosol (Figure 3B). The variations in molecular function (MF) were markedly enriched in actin filament binding, structural constituent of muscle, and actin binding (Figure 3C). Analysis of the KEGG pathway revealed that the all DEGs were primarily enriched in dilated cardiomyopathy, adrenergic signaling in cardiomyocytes, and hypertrophic cardiomyopathy (HCM) (Figure 3D).

GSEA was used to perform GO and KEGG analysis to explore the function and pathways of DEGs. GO enrichment analysis show that 2710/4412 gene sets were upregulated in atherosclerosis, 822 gene sets were significantly enriched at nominal p-value < 0.05, and 235 gene sets are significantly enriched at nominal p-value < 0.01. In addition, 1702/4412 gene sets are downregulated in atherosclerosis, 308 gene sets are significantly enriched at nominal p value <5%, and 89 gene sets are significantly enriched at nominal p-value < 0.01. The enrichments for both up- and down-regulated gene sets in the significant order (size of NES) are listed in Table 1, such as “GO_SKELETAL_MUSCLE_ CONTRACTION”, “GO_NEGATIVE_REGULATION _OF_SMOOTH_MUSCLE_CONTRACTION”, “GO_ ACTIN_FILAMENT_POLYMERIZATION”, “GO_ ACTIN_FILAMENT_BUNDLE_ORGANIZATION”, “GO_SMOOTH_MUSCLE_CONTRACTION”, and “GO_MUSCLE_CONTRACTION”. GSEA also revealed that upregulated gene sets in atherosclerosis were mainly associated with muscle contraction, and actin filament. Furthermore, KEGG enrichment analysis indicated that 121/174 gene sets are upregulated in atherosclerosis compared to normal artery samples, 40 gene sets are significantly enriched at nominal p value <5%, and 16 gene sets are significantly enriched at nominal p-value < 1%. 53/174 gene sets are downregulated in atherosclerosis, 10 gene set is significantly enriched at nominal p value <5%, and 2 gene sets are significantly enriched at nominal p-value < 1%. We respectively displayed important 9 gene sets correlated with atherosclerosis according to NES in Table 2, such as “KEGG_EPITHELIAL_CELL_ SIGNALING_IN_HELICOBACTER_PYLORI_INFECTION”, “KEGG_BASE_EXCISION_REPAIR”, “KEGG_ DILATED_CARDIOMYOPATHY”, and so on.

**Table 1:** Functional enrichment analysis of DEGs in atherosclerosis using GSEA.

**Gene set name**	**SIZE**	**ES**	**NES**	**Rank at max**
**Upregulated**				
GO_SKELETAL_MUSCLE_CONTRACTION	29	0.316	0.939	2942
GO_NEGATIVE_REGULATION_OF_SMOOTH_MUSCLE_CONTRACTION	15	0.360	0.902	3373
GO_ACTIN_FILAMENT_POLYMERIZATION	20	0.393	1.205	2007
GO_ACTIN_FILAMENT_BUNDLE_ORGANIZATION	47	0.302	1.064	1726
GO_CYTOSOLIC_CALCIUM_ION_TRANSPORT	48	0.435	1.218	1988
GO_CYTOSOLIC_PART	192	0.256	1.011	3378
**Downregulated**				
GO_SMOOTH_MUSCLE_CONTRACTION	44	−0.580	−1.577	2784
GO_MUSCLE_CONTRACTION	220	−0.514	−1.573	2897
GO_REGULATION_OF_SMOOTH_MUSCLE_CONTRACTION	60	−0.505	−1.422	2121
GO_ACTIN_FILAMENT_ORGANIZATION	158	−0.308	−1.195	2174
GO_CYTOSOLIC_TRANSPORT	188	−0.237	−1.144	2287
GO_ENDOPLASMIC_RETICULUM_TO_CYTOSOL_TRANSPORT	21	−0.347	−1.104	27

ES: Enrichment Score; NES: Normalized Enrichment Score.

**Table 2:** Pathway enrichment analysis of DEGs in atherosclerosis using GSEA.

**Gene set name**	**SIZE**	**ES**	**NES**	**Rank at max**
**Upregulated**				
KEGG_EPITHELIAL_CELL_SIGNALING_IN_HELICOBACTER_ PYLORI_INFECTION	61	0.563	1.525	3156
KEGG_BASE_EXCISION_REPAIR	31	0.459	1.522	6082
KEGG_ANTIGEN_PROCESSING_AND_PRESENTATION	57	0.653	1.519	2554
KEGG_T_CELL_RECEPTOR_SIGNALING_PATHWAY	101	0.605	1.510	3648
KEGG_TOLL_LIKE_RECEPTOR_SIGNALING_PATHWAY	88	0.702	1.508	2235
KEGG_PATHOGENIC_ESCHERICHIA_COLI_INFECTION	45	0.499	1.505	4454
**Downregulated**				
KEGG_DILATED_CARDIOMYOPATHY	86	−0.524	−1.434	1865
KEGG_HYPERTROPHIC_CARDIOMYOPATHY_HCM	80	−0.499	−1.407	2006
KEGG_LONG_TERM_POTENTIATION	65	−0.390	−1.300	1772
KEGG_ADHERENS_JUNCTION	72	−0.425	−1.272	2780

ES: Enrichment Score; NES: Normalized Enrichment Score.

What’s more, the enrichment analysis of metascape also demonstrates that the DEGs between control and atherosclerosis were markedly enriched in the actin filament-based process (Figure 3E–3G).

### PPI network construction, module analysis, hub gene selection and analysis

**Figure 4:**
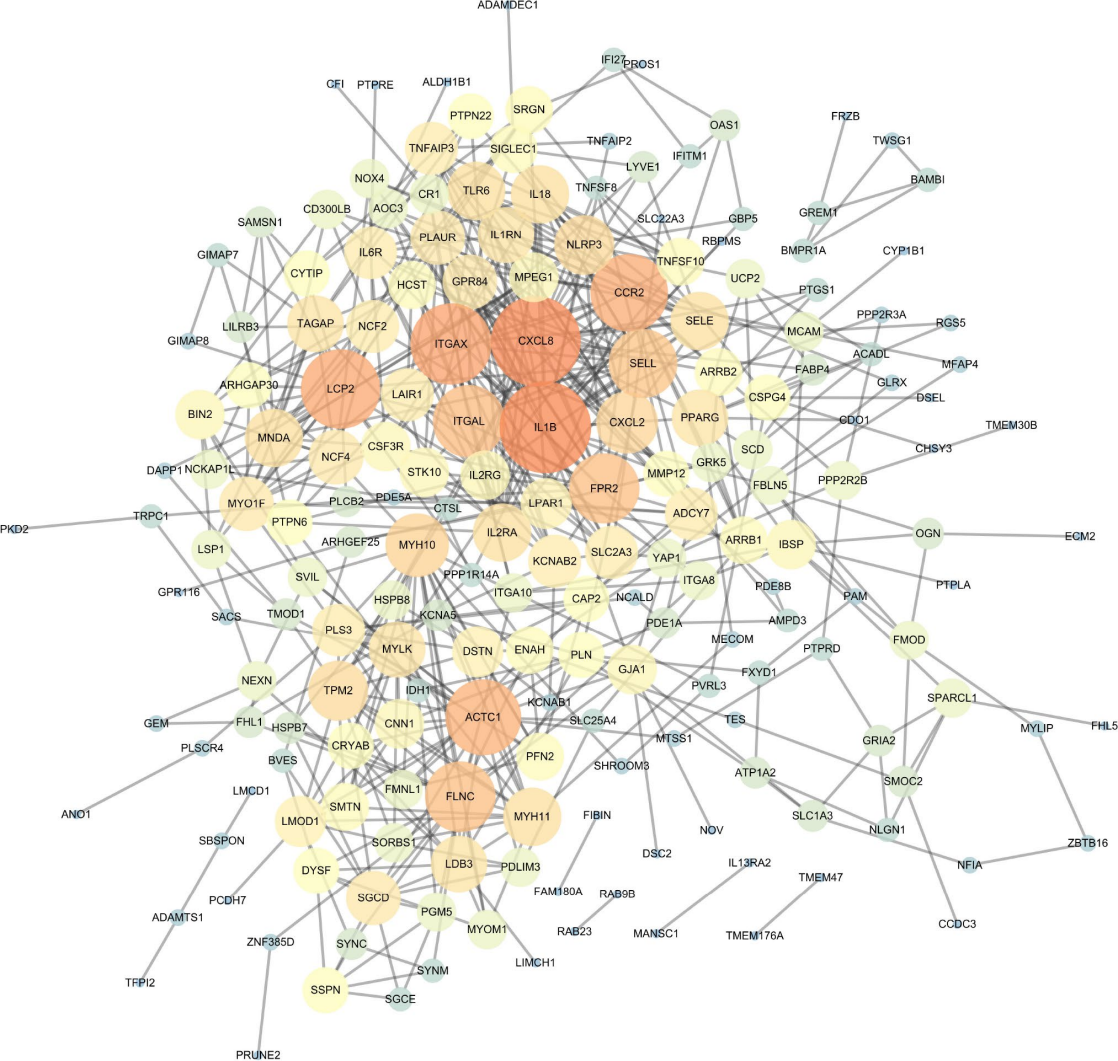
**The protein-protein interaction (PPI) network of differentially expressed genes (DEGs).**

**Figure 5:**
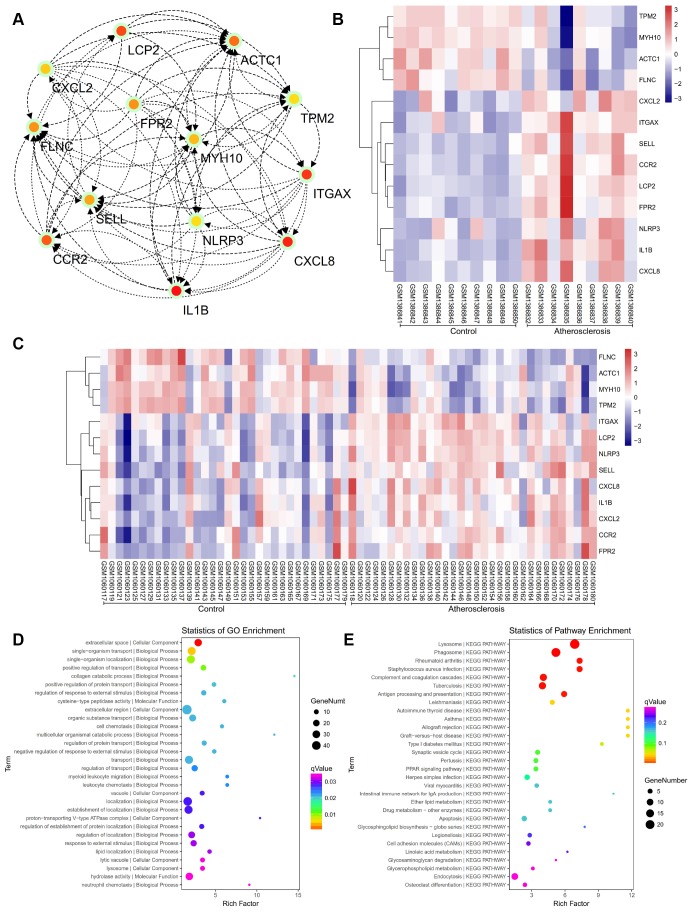
(**A**) The hub genes were identified from the PPI network. (**B**, **C**) Hierarchical clustering demonstrated that the hub genes could effectively differentiate the atherosclerotic samples from the non-atherosclerotic samples in the GSE57691 and GSE43292 datasets. The upregulated genes are marked in pink, the downregulated genes are marked in blue. (**D**) The GO enrichment analyses of DEGs of our private dataset. (**E**) The KEGG pathway analysis of DEGs of our private dataset.

The PPI network of DEGs was constructed (Figure 4) and the most significant module and network of hub genes was identified using Cytoscape software (Figure 5A). A total of 13 genes were identified as hub genes with a degree of ≥13. Hierarchical clustering demonstrated that the hub genes could effectively differentiate the atherosclerotic samples from the non-atherosclerotic samples (Figure 5B and 5C). Among these genes, MYH10, ACTC1, FLNC and TPM2 exhibited the lower expression in the atherosclerosis samples, while the expressions of CXCL2, ITGAX, SELL, CCR2, LCP2, FPR2, NLRP3, IL1B, and CXCL8 were up-regulated in the atherosclerosis samples, suggesting that they may exert pivotal functions in the occurrence or progression of atherosclerosis.

### Verification of the animal model and proteomics

**Figure 6:**
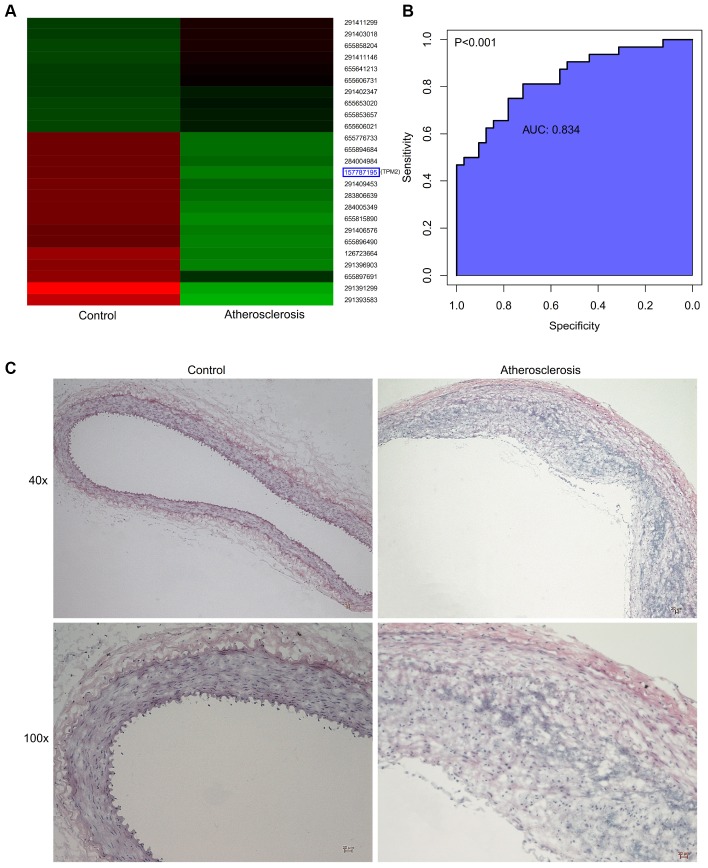
(**A**) Hierarchical clustering revealed the expression situation of DEGs between control and atherosclerotic samples in our private dataset. Upregulated genes are marked in red, downregulated genes are marked in green. The right legends represent Protein ID, where “157787195” represents TPM2. (**B**) The receiver operator characteristic curve, indicating that the expression level of TPM2 in the GSE43292 could predict atherosclerosis sensitively and specifically. (**C**) The pathological observation of artery of the control and atherosclerosis groups through the HE staining. (Gross appearance, 40x, 100x). HE: hematoxylin-eosin.

After the construction of animal model, proteomic analysis was performed. The results of GO analysis of DEGs from our animal model exhibited the most similarity with the GSE43292 and GSE57691 datasets. The detailed information of changes in the BP, CC, and MF, are presented in the Figure 5D. KEGG pathway analysis for our own dataset displayed that DEGs were primarily enriched in the lysosome, phagosome, rheumatoid arthritis, staphylococcus aureus infection, and so on. (Figure 5E). Hierarchical clustering presented in Figure 6A revealed the expression situation of DEGs between control and atherosclerotic samples in our private dataset. The upregulated genes are marked in red, and the downregulated genes are marked in green. The right legends of Figure 6A represent Protein ID, and the “157787195” presents TPM2.

### Common DEG between public and private datasets

According to comparison between public and private datasets, the common DEG was identified, which is TPM2. The present study identified that TPM2 in the atherosclerotic tissues was displayed at significantly lower levels when compared with the matched normal tissues (P<0.05). The receiver operator characteristic curve indicates that the expression level of TPM2 in the GSE43292 could predict atherosclerosis sensitively and specifically (area under the curve for intimal thickness, 0.837; p<0.001) (Figure 6B).

### Association between TPM2 and atherosclerosis by Pearson correlation test and univariate linear regression

Pearson correlation coefficient displayed that the status of atherosclerosis was significantly correlated with the expression of TPM2 (ρ=-0.567, p<0.001). The natural logarithmic atherosclerosis remained related to the TPM2 (β=-0.461 p<0.001) in the univariate linear regression model (Table 3).

**Table 3:** The correlation and linear regression analysis between atherosclerosis and relevant gene expression.

**Gene symbol**	**Atherosclerosis**				
	**Pearson’s correlation coefficient**			**Univariate linear regression**	
	**ρ^a^**	**p-value**		**β^b^**	**P-value**
TPM2	‒0.567	<0.001*		‒0.461	<0.001*
MYH10	‒0.512	<0.001*		‒0.329	<0.001*
ACTA1	‒0.503	<0.001*		‒0.236	<0.001*
IL1B	0.406	0.001*		0.211	0.001*
ITGAX	0.518	<0.001*		0.250	<0.001*
SELL	0.504	<0.001*		0.422	<0.001*
CCR2	0.400	0.001*		0.241	0.001*
LCP2	0.576	<0.001*		0.405	<0.001*
CXCL8	0.373	0.002*		0.149	0.002*
NLRP3	0.584	<0.001*		0.480	<0.001*
FLNC	‒0.575	<0.001*		‒0.430	<0.001*
CXCL2	0.358	0.004*		0.213	0.004*
FPR2	0.383	0.002*		0.206	0.002*

^a^Pearson’s correlation coefficient between atherosclerosis and relevant characteristics; ρ: Pearson’s correlation coefficient.

^b^Univariate linear regression analysis, β: parameter estimate.

*Significant variables: P<0.05

### An independent risk factor for atherosclerosis based on univariate and multivariate logistic regression: TPM2

Table 4 presents the univariate odd ratios (ORs) and 95% confidence intervals (95%CI) for atherosclerosis of hub genes. The OR for atherosclerosis was 0.090 (95% CI, 0.028-0.293, p<0.001) in the group with high expression of TPM2 compared with low expression of TPM2 (Table 4). In order to effectively control the influence of confounding factors, multivariate factors were incorporated into the multivariate logistic regression model simultaneously, which can also predict the most independent risk characteristic. Table 5 showed the result of multivariate logistic proportional regression analysis. The higher expression of TPM2 has, the significantly greater benefit there has, and the OR of high TPM2 is 0.140 (95% CI, 0.020-0.988; p=0.049) (Table 5).

**Table 4:** Correlative parameters’ effect on atherosclerosis based on univariate logistic proportional regression analysis.

**Parameters**			**AS**		
			**OR**	**95% CI**	**P**
TPM2	Low	29	1		<0.001*
	High	35	0.090	0.028–0.293	
MYH10	Low	26	1		0.001*
	High	38	0.138	0.044–0.433	
ACTC1	Low	35	1		<0.001*
	High	29	0.127	0.041–0.391	
IL1B	Low	29	1		0.026*
	High	35	3.215	1.150–8.987	
ITGAX	Low	27	1		<0.001*
	High	37	8.273	2.622–26.100	
SELL	Low	30	1		<0.001*
	High	34	13.000	3.935–42.950	
CCR2	Low	33	1		0.007*
	High	31	4.200	1.478–11.936	
LCP2	Low	24	1		0.001*
	High	40	7.892	2.409–25.857	
CXCL8	Low	33	1		0.026*
	High	31	3.182	1.145–8.841	
NLRP3	Low	29	1		<0.001
	High	35	11.074	3.418–35.878	
FLNC	Low	35	1		<0.001*
	High	29	0.062	0.018–0.214	
CXCL2	Low	34	1		0.048*
	High	30	2.790	1.011–7.698	
FPR2	Low	39	1		0.006*
	High	25	4.592	1.542–13.671	

OR, odds ratio; 95% CI, 95% confidence interval. * P < 0.05

**Table 5:** Correlative genes’ effect on AS based on multiple logistic proportional regression analysis.

**Genes**		**AS**	
	**OR**	**95% CI**	**P**
TPM2	0.140	0.020–0.988	0.049*
IL1B	1.105	0.181–6.730	0.914
CCR2	0.819	0.152–4.419	0.817
CXCL8	1.142	0.125–10.477	0.906
CXCL2	0.486	0.046–5.171	0.550
FPR2	2.658	0.467–15.111	0.270
NLRP3	6.032	0.880–41.363	0.067
MYH10	2.611	0.222–30.678	0.445

AS: atherosclerosis; OR, odds ratio; 95% CI, 95% confidence interval. * P < 0.05

### Effects of the atherosclerosis on the abdominal aorta in the aspect of intima-media thickness and smooth muscle cells

**Figure 7:**
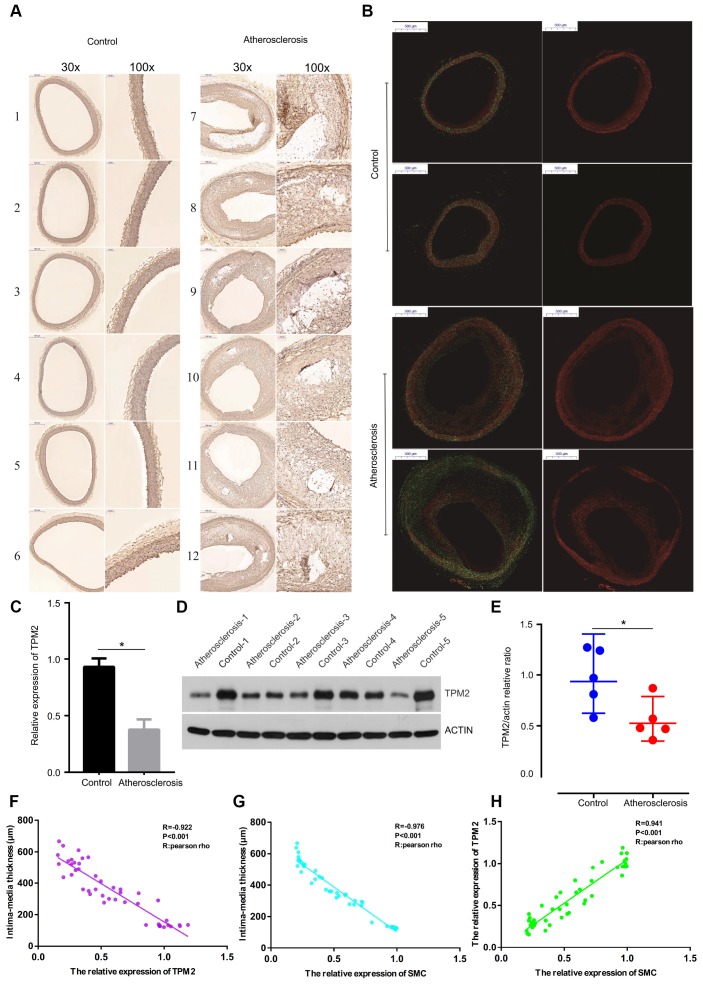
(**A**) The detection of SMC of the artery wall by immunohistochemical staining with anti-SMC monoclonal antibody. (Gross appearance, 30x, 100x). (**B**) The detection of SMC content of the artery wall by immunofluorescence assay. (Gross appearance, 30x). (**C**) Relative expression of TPM2 by RT-qPCR analysis. *p< 0.05, compared with control. (**D**) Western blotting expression of TPM2 in the control (Con) and atherosclerosis (AS) groups. (**E**) Quantitative comparison of TPM2 expression between the two groups. (**F**) The linear correlation between intima-media thickness and the relative expression of TPM2. (**G**) The linear correlation between intima-media thickness and the relative expression of SMC. (**H**) The linear correlation between the relative expression of TPM2 and the relative expression of SMC. SMC: smooth muscle cell.

Figure 6C presents the status of abdominal aorta from the microcosmic appearances, which showed that intima-media thickness was incrassated in the atherosclerosis group, compared with the control group (Figure 6C). Through the immunohistochemical staining (Figure 7A) and immunofluorescence assay (Figure 7B), the artery wall of the atherosclerosis group was more deficient in smooth muscle cells than the control group.

### Verification of the expression of TPM2

According to the above expression analysis, TPM2 were markedly down-regulated in atherosclerosis samples. Results of RT-qPCR and western blotting analysis showed that the relative expression level of TPM2 was significantly lower in atherosclerosis samples, compared with the control groups (p<0.05) (Figure 7C–7E). The result demonstrated that TPM2 might be considered as biomarkers for atherosclerosis.

### Strong associations between the intima-media thickness, the relative expression of TPM2 and SMC

**Figure 8:**
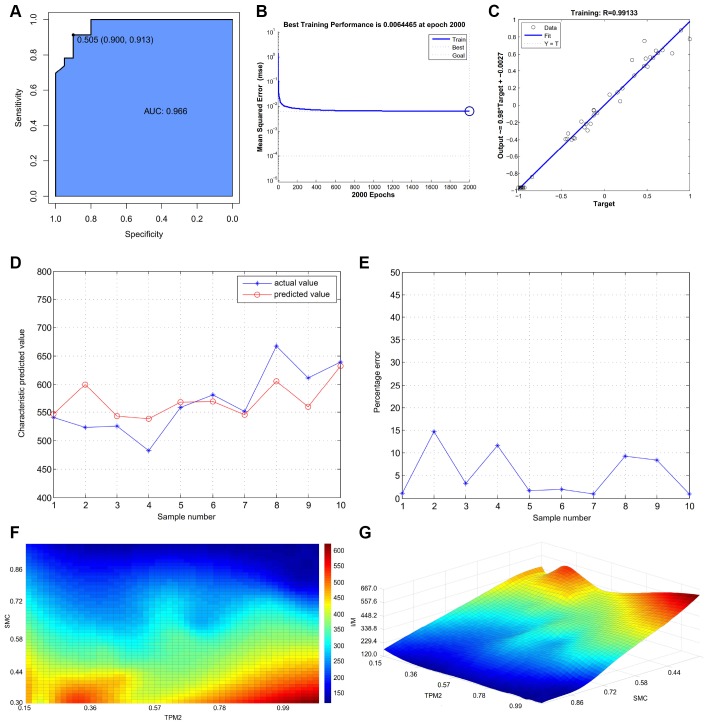
(**A**) The receiver operator characteristic curve, indicating that the expression level of TPM2 in the own experiment could predict atherosclerosis sensitively and specifically. (**B**) The neural network prediction model of atherosclerosis intima-media thickness. The best training performance is 0.0064465 at epoch 2000. (**C**) The final training model of neural network prediction model, and the relativity is 0.99133. (**D**) Verify the predicted value of the data against the actual value. (**E**) Verify the data error percentage curve. (**F**) The high-risk warning range of atherosclerosis intima-media thickness at the level of the planform. (**G**) The high-risk warning range of atherosclerosis intima-media thickness at the level of the three dimensional stereogram. The color represents the intima-media thickness: the “red” represents “high”, the “blue” represents “low”.

The intima-media thickness was negatively associated with the relative expression of TPM2 (Pearson Rho=-0.922, P<0.001) (Figure 7F), and the relative expression of SMC (Pearson Rho=-0.976, P<0.001) (Figure 7G). And there is a positive association between the relative expression of TPM2 and the relative expression of SMC (Pearson Rho=0.941, P<0.001) (Figure 7H). The receiver operator characteristic curve indicates that the expression level of TPM2 in our own experiment also could predict atherosclerosis sensitively and specifically (area under the curve for intimal thickness, 0.966; p<0.05) (Figure 8A).

### The neural network prediction model and high-risk warning range of atherosclerosis intima-media thickness

After training, the neural network prediction model reached the best effect, in which mean squared error is 0.0064465 at epoch 2000 (Figure 8B), and the relativity is 0.99133 (Figure 8C). Through verifying the predicted value of the data against the actual value, we found that there are only small differences (Figure 8D, 8E). Based on the above result, we could speculate that the expression of TPM2 and smooth muscle cell might be the predictive indexes of atherosclerosis intima-media thickness.

Through the cubic spline interpolation algorithm, we find the high-risk warning indicator of atherosclerosis intima-media thickness: 0.226 < TPM2 < 0.455, and 0.3 < expression of smooth muscle cell< 0.44 (Figure 8F). Furthermore, the three dimensional stereogram could present the warning range well (Figure 8G).

## DISCUSSION

Due to the health complications associated with aging, the incidence of ischemic cardiovascular and cerebrovascular diseases is increasing, and is now the leading cause of disability and mortality worldwide recently [14, 15]. Atherosclerosis is a chronic inflammatory disease, that can be induced by a plethora of factors, including environment, diet, polygenetic inheritance among other factors [16]. Atherosclerosis is the principal pathological cause for coronary heart disease, myocardial infarction, cerebral infarction, cerebral hemorrhage and other cardiovascular and cerebrovascular diseases [17]. Previous studies [18, 19] have demonstrated that autophagy and p62 serve a significant role in the development of atherosclerosis. In the early stage of atherosclerosis, an appropriate increase in the expression of p62 and its ubiquitination may reinforce the stability of plaques and delay the development of atherosclerosis. In the middle and late stage of atherosclerosis, the process of autophagy has been revealed to impede p62 aggregation, inhibit relevant pathways, and attenuate plaque instability [20]. Activated NOD-like receptor family pyrin domain containing 3 (NLRP3) inflammasome was observed to transform caspase-1 precursor into active caspase-1, promote the maturation and secretion of interleukin-1 beta and IL-18, and then trigger the inflammatory response of the body [21]. NLRP3 is closely associated with the formation of atherosclerosis. A large proportion of patients with atherosclerosis are not diagnosed in the early stage of disease, and therefore are not appropriate candidates for treatment, which is one of the principal causes for unfavorable patient outcomes. Therefore, the identification and investigation of underlying biomarkers for high-efficiency therapy and diagnosis are urgently required. Bioinformatics technology permits researchers to probe and investigate the genetic changes in atherosclerosis, and has been demonstrated to be a valid method to distinguish novel biomarkers in other diseases [9–12].

Following the analysis of the 2 microarray datasets in the current research, DEGs between atherosclerotic and non-atherosclerotic tissues were identified. A total of 234 DEGs were contained in the 2 datasets simultaneously, and the DEGs’ interactions were explored via KEGG and GO analyses, The DEGs were primarily enriched in actin filament, actin binding, smooth muscle cells, and cytokine-cytokine receptor interactions. Previous researches have reported that the maladjustment of cellular process and cellular component organization or biogenesis has a substantial impact on the occurrence and development of atherosclerosis [22–24]. In addition, recent research has hypothesized that atherosclerosis exerts an accelerating role for actin binding and catalytic activity [25]. In addition, cytokine-cytokine receptor interactions and cell adhesion molecules have also been reported to serve an important role in the stability of plaque formation which is frequently altered in atherosclerosis [26, 27]. In brief, the results presented herein are consistent with the aforementioned studies.

Following the analysis of the public and private datasets, the expression of TPM2 in the atherosclerotic tissues was observed to be lower than in normal tissues. TPM2 is a subtype of tropomyosin [28]. Tropomyosin (TPM), a thin filament-associated protein, and has been hypothesized to serve a crucial role in the regulation of muscle contraction [29]. TMP is widely distributed in various eukaryotic cells in the form of a large number of isomers, and four gene subtypes of TPM have been identified in mammals, which are respectively named TPM1, TPM2, TPM3 and TPM4 [30]. TPM is associated with cellular migration, morphogenesis and the regulation of actin filaments [28]. TPM2 is located on chromosome 9p13.2-p13.1, and the CDS sequence is 1120bp, and encoding protein mass is 36000. TPM2 contains 11 exons in rats, among which exons 3, 4, 5, 7 and 8 are expressed in all known isomers. The gene encodes, in addition to skeletal muscle beta TPM, TPM1 isomers of smooth muscle beta TPM and fibroblasts. The gene encodes not only β-TPM of skeletal muscle, but also β-TPM of smooth muscle and TPM1 isomers of fibroblasts. Despite sequence differences, all the β-TPM isomers are composed of 284 amino acids. The chicken beta gene encodes a type of fibroblast, TPM-3b [31].

TPM regulates the dynamic changes of actin filaments in the cell migration, morphogenesis, and cytoplasmic migration [32]. The study of Cui et al. discovered that TPM2 appears to be commonly silenced by aberrant DNA methylation in colon cancer. TPM2 loss is associated with Ras homolog gene family member A (RhoA) activation and tumor proliferation and transfer [33]. In addition, the study of Zhang et al. identified that TPM2 expression is downregulated in breast cancer cells when compared with that in normal breast cells [28]. In the process of tumor cell metastasis, cancer cells break away from tumor tissue and move to target organs. These processes are inseparable from cell movement [34]. Therefore, TPM2 may also be involved in the process of tumor formation and metastasis via this mechanism. In addition, we hypothesize that TPM2 is closely associated with the process of cell formation and metastasis in atherosclerotic tissue.

Clarkson [35] found that TPM2, actin, and troponin are mainly related to congenital myopathy. Bailey’s research [36] demonstrated that TPM is the molecular target leading to high-temperature superconducting (HTS) efforts. Bartelt [37] found that HTS could prevent the formation and development of atherosclerosis. On the one hand, HTS can reduce residual lipoproteins that are involved in atherosclerosis. On the other hand, HTS can also improve high-density lipoprotein (HDL) remodeling and promote cholesterol clearance. Moreover, in terms of lipid deposition, HTS can also increase the apoptosis of macrophages and reduce the formation of foam cells. What’s more, endothelial function remains an important role in the formation of atherosclerosis. Abnormal expression of myosin microfilaments could damage endothelial cells, provide a binding point for lipids and calcium, and also stimulate fibroblast proliferation and exacerbates fibrosis. However, the role of the TPM family is to stabilize myosin filaments and endothelial function [38]. This suggests that the low expression of TPM2 in the diseased arteries might damage the function of endothelial cells, and lead to the occurrence of atherosclerosis. Also, abnormal function of smooth muscle cell is still a factor causing atherosclerosis. TPM could maintain the stability of actin of smooth muscle, and indirectly promote the vasoconstriction function. Therefore, the lack of TPM might limit vasoconstriction [39]. Excessive dilation of blood vessels will lead to rupture and degeneration of the middle artery, which will induce the formation of atherosclerosis [40].

Previous studies have demonstrated that in atherosclerotic tissue, the number of vascular smooth muscle cells in the middle membrane is decreased, the middle membrane becomes thinner, and the intimal plaque is rich in macrophages [41]. In addition, the results of the present study revealed that the expression of TPM2 in atherosclerotic tissues was significantly reduced, and TPM2, as one subtype of TPM, was abundant in skeletal muscle, smooth muscle and fibroblasts. TPM2 is also involved in muscle contraction, cell movement and other biological process [28, 29, 31]. This has culminated in the hypothesis that, in the development of atherosclerosis, the down-expression of TPM2 leads to disorders in the formation and movement of vascular smooth muscle cells. The middle membrane of blood vessel subsequently becomes thinner and brittle, which could cause the weak contractility. Subsequently, macrophages may phagocytose ineffective smooth muscle cells or fragile fibroblasts of the vascular middle membrane, and migrate to the vascular intima to participate in the formation of atherosclerotic plaques.

### Limitations

Despite the rigorous bioinformatics analysis in this study, there are still some limitations. First, the sample size of our study is small, which might result in some deviations in the results. Secondly, we only conducted bioinformatics mining and animal experimental verification, which could demonstrate that TPM2 plays an important role in the occurrence and development of atherosclerosis to some extent. However, there are still differences between animals and human. Currently, it is difficult to cut vascular tissue of healthy human, which refers to the ethical issue and informed consent. Therefore, the research has not obtained the evidence from human.

### Future directions

In the next stage of research, we will try to obtain the ethical approval documents and informed consent so that the further study could perform verification from the human. And multicenter randomized controlled clinical trial should be performed, and recruit more subjects to verify the relationship between expression level of TPM2 and formation of atherosclerosis.

In the present research, a total of 234 DEGs and 13 hub genes were identified and authenticated between atherosclerotic tissues and non-atherosclerotic tissues. Following verification of animal model, the common DEG, Tropomyosin 2 (TPM2), was found, which were displayed at lower levels in the atherosclerosis models and samples. In summary, DEGs identified in the present study may assist clinicians in understanding the pathogenesis governing the occurrence and development of atherosclerosis, and TPM2 exhibits potential as a promising diagnostic and therapeutic biomarker for atherosclerosis.

## METHODS

### Access to public data

GEO (http://www.ncbi.nlm.nih.gov/geo) [42] is an open functional genomics database of high-throughput resource that includes microarrays, gene expression data and chips. Two expression profiling datasets [GSE43292 [43] and GSE57691 [44]], which all identify genes and pathways involved in the formation of atherosclerotic plaques from normal individuals, were obtained from the GEO (GPL6244 platform, [HuGene-1_0-st] Affymetrix Human Gene 1.0 ST Array [transcript (gene) version], and GPL10558 platform, Illumina HumanHT-12 V4.0 expression beadchip). The probes were transformed into the homologous gene symbol by means of the platform’s annotation information. The GSE43292 dataset contained 32 non-atherosclerotic arterial tissues samples and 32 atherosclerotic arterial tissues samples. The GSE57691 dataset contained 10 non-atherosclerotic arterial tissues samples and 9 atherosclerotic arterial tissues samples.

### Intra-group data repeatability test

The Pearson’s correlation test was performed to verify intra-group data repeatability in the per group. The R programming language was used to provide the software and operating environment for statistical analysis and drawing of graphs. Correlations between all samples from the same dataset were visualized using heat maps which were also drawn using R. Principal component analysis (PCA) is a commonly used method for sample clustering, and is often used for gene expression, diversity analysis, resequencing, and other sample clustering based on various variable information. The intra-group data repeatability of the dataset was tested by sample clustering analysis.

### DEGs identified by GEO2R

The GEO2R (http://www.ncbi.nlm.nih.gov/geo/geo2r), is an online data analysis tool, and was used to screen the DEGs between non-atherosclerotic and atherosclerotic tissues. After establishing the differential experimental groups for one GEO series, GEO2R could execute a command to compare the differential classifications so that the DEGs would be identified. When the gene symbol corresponded to probe sets, the data was considered valuable and would be reserved. The values for statistical significance were set as P-value≤0.01 and Fold change (FC)≥1.5 [45]. Volcano maps were drawn using the volcano plotting tool (https://shengxin.ren). The DEGs were then screened by introducing the two datasets into the FunRich (functional enrichment analysis tool) (http://www.funrich.org/). Venn diagrams were delineated using an online Venn tool (http://bioinformatics.psb.ugent.be/webtools/Venn/), which could then be used to visualize common DEGs shared between the two datasets. The overlaps between differently expressed gene lists are performed by the Circos [46]. Another useful representation is to overlap genes based on their functions or shared pathways. The overlaps between gene lists can be significantly improved by considering overlaps between genes sharing the same enriched ontology term. Only ontology terms that contain less than 100 genes are used to calculate functional overlaps to avoid linking genes using very general annotation.

### Construction of weighted gene co-expression network analysis (WGCNA)

The R package (version 3.5.0) was used to preprocess and normalize the raw data of GSE43292. Ranked by P-value from small to large (including control and atherosclerosis samples), a total of 936 DEGs in the GSE43292 were chosen for WGCNA. WGCNA is a systematic biological method used to describe the gene association modes among different samples, and it can be used to identify gene sets with highly synergistic changes, and identify alternate biomarkers or therapeutic targets based on the coherence of gene sets and the correlation between gene sets and phenotypes. The WGCNA could use thousands of the most varied genetic information to identify the sets of genes of interest and to conduct significant association analysis with phenotypes. The procedure of WGCNA consists of four steps, including (a) identification of modules: hierarchical clustering analysis was conducted based on weighted correlation, and different gene modules were obtained according to the results of segmentation and clustering according to the set standards, which were represented by branches and different colors of the cluster tree; (b) construction of the gene co-expression network; (c) studying module relationships; (d) calculation of the correlation between gene modules and phenotypes, and the modules related to clinical traits were identified

### Functional annotation of DEGs by DAVID and metascape analyses

DAVID (https://david.ncifcrf.gov/home.jsp) (version 6.8), is an online analysis tool suite with the function of Integrated Discovery and Annotation, that provides typical batch annotation and gene-Gene Ontology (GO) term enrichment analysis to highlight the most relevant Gene Ontology (GO) terms associated with a given gene list [47–49]. The Kyoto Encyclopedia of Genes and Genomes (KEGG) (https://www.kegg.jp/) is one of the most commonly used biological information databases worldwide, and aims to understand advanced functions and biological systems. At a molecular level, KEGG integrates large numbers of practical program database resources from high-throughput experimental technologies [50]. GO is an ontology database widely used in bioinformatics, which covers three aspects of biology, including cellular components, molecular functions and biological processes [51]. Finally, OmicShare (http://www.omicshare.com/tools), an open data analysis platform, was used to visualize the enrichment analysis [52]. The metascape.org (http://metascape.org/gp/index.html#/main/step1) was used to perform enrichment analysis for DEGs again. P<0.05 was considered to indicate a statistically significant difference.

### Enrichment analysis by Gene Set Enrichment Analysis (GSEA)

Gene Set Enrichment Analysis (GSEA) could analyze all sequenced genes in two sample groups, its input is a gene expression matrix in which genes are divided into two groups, and all genes are first sequenced and then used to indicate the changing trend of expression level of genes between the two groups. GSEA analyzes whether all genes of a priori defined set are enriched at the top or bottom of this sequence list. GO and KEGG pathway enrichment analysis were performed for identified DEGs using GSEA analysis. GSEA analysis was conducted on all sequenced genes of atherosclerosis tissues and normal artery tissues through GSEA software, after importing gene annotation files, reference function sets, and all gene data of both atherosclerosis tissues and normal artery tissues, the software would perform analysis and sequence the genes according to algorithm, thus a gene sequence list is obtained, then it will analyze position of all genes in the sequence list and accumulate them to get the enrichment score (ES), after standardizing the ES, we could get a comprehensive understanding of biological function of gene through enrichment of function sets. P<0.05 was set as the cut-off criterion.

### Construction and analysis of protein-protein interaction (PPI) network and the analysis and mining of hub genes

After importing the common DEGs into the Search Tool for the Retrieval of Interacting Genes (STRING; http://string-db.org) (version 10.5) [53], the online tool predicted and outlined the PPI network. The analysis of interactions between various proteins provide novel hypothesizes for the pathophysiological mechanisms governing the development of AS. In the present study the STRING database was used to construct the PPI network of DEGs, and the minimum required interaction score was a medium confidence of >0.4 [45]. The score > 0.4 is an important restriction of the network. If the rule of score changed, the PPI maybe different. Cytoscape (version 3.6.1) is a free visualization software and was applied to visualize the PPI networks [54, 55]. Based on the topology principles, the Molecular Complex Detection (MCODE) (version 1.5.1), a plug-in of Cytoscape, could discover the tightly coupled region [56]. The Cytoscape software initially constructed the PPI network map. Secondly, MCODE identified the most important module of the network map. The criteria of MCODE analysis was a degree cut-off=2, MCODE scores >5, Max depth=100, k-score=2, and node score cut-off=0.2. When the degrees were set (degrees≥13) [45], the hub genes were excavated. The clustering analysis of hub genes was performed using OmicShare.

### The animal model construction

A total of 20, 3-month-old New Zealand white rabbits were acquired from the Institute of Laboratory Animal Sciences, Chinese Academy of Medical Sciences (CAMS) & Peking Union Medical College. The rabbits were randomly divided into two groups. In the control group (CON group, n=10), rabbits were fed with standard rabbit chow (0% cholesterol), and did not undergo abdominal aortic balloon injury. In the atherosclerosis group (n=10), the rabbits were fed a high fat diet (6% bean oil and 1% cholesterol) for eight weeks, and underwent abdominal aortic balloon injury. The Animal Care and Use Committee of the Institute of Laboratory Animal Sciences, Chinese Academy of Medical Sciences and Peking Union Medical College (CAMS∆PUMC) authorized the experimental ethics agreement.

With fasting 12h before surgery and unlimited drinking, the rabbits of atherosclerosis group were operated after anesthesia with 3% sodium pentobarbital solution (3mg/kg of animal body weight; provided by institute of laboratory animal sciences, CAMS&PUMC, Beijing, China). Then, along the right femoral artery, the surgeon opened the skin, separated the subcutaneous tissue layer by layer and freed the femoral artery(2-3cm). Furthermore, punctured the femoral artery, put in a 4F vessel sheath, expanded and pulled back the balloon for 3 times, ligatured the right femoral artery, and sutured skin. Finally, animals were taken 40,000 U penicillin after surgery for 5 days.

Following euthanasia of rabbits with 3% pentobarbital solution (300mg/kg of animal body weight; provided by institute of laboratory animal sciences, CAMS∆PUMC, Beijing, China) and air embolization, and the indicators of judging death include breathing and cardiac arrest, pupil dilation, and nerve reflex disappeared. Then, the abdominal aortic tissues were dissected. The establishment of model animals is groping for ourselves. To evaluate atherosclerosis model, gross examination of abdominal aorta was performed by HE staining.

### Proteomic analysis

Rabbit abdominal aortic tissues were ground in liquid nitrogen and lysed by protein extraction buffer (8M urea, 0.1%SDS) containing additional protease inhibitor cocktail (Roche, Switzerland) and 1mM phenylmethylsulfonyl fluoride (Beyotime Biotechnology, China) on ice for 30 min and then centrifuged at 14,000 rpm for 15 min at 4°C. The supernatant was gathered and the proteins’ concentration was measured with BCA assay (Pierce, USA). The cell lysis was stored at -80 degrees Celsius before further processing. Samples are tested for quality by using BCA quantitative kit. iTRAQ (AB Sciex, USA) with different reporter ions (113-121 Da) were applied as isobaric tags for relative quantification. iTRAQ labeling was conducted on the basis of the instructions of manufacturer. C18 chromatographic column sample classification was carried out. A total of 40 fractionations of labeled peptides were further concatenated into 20 fractions, vacuum dried and deposited at -80 degrees Celsius until further LC-MS analysis. And the LC-MS/MS analysis was performed by Q Exactive mass spectrometer (Thermo Scientific, USA). The NCBI s RefSeq rabbit protein sequence database was searched by the Sequest algorithms with Proteome discoverer software (version 1.4) (Thermo Scientific, USA). And the DEGs were identified in our own data. The rule for statistical significance was that adj. P-value≤0.01 and Fold change (FC) ≥1.5.

### The level of smooth muscle cell (SMC)

Paraffin sections were made using the tissue samples of the abdominal artery. The paraffin sections were deparaffinized with water, sealed with hydrogen peroxide, then washed with double distilled water. SMC were detected by using immunohistochemistry after antigen retrieval. Anti-SMC monoclonal antibody was used to detect macrophagocytes. According to the instructions of the VECTASTAIN Elite ABC Kit (Vector Laboratories, Burlingame, CA, USA), the specific detection steps were performed as follows. First, antigen-fixed paraffin sections were washed with phosphate-buffered saline (PBS) two to three times (5 min/times) and blocked with 10% goat serum (TransGen Biotech, Beijing, China) at 37°C for 20minutes. Second, removed the serum by using filter paper, and added YAP or TAZ Rabbit polyclonal antibody (Abcam, Cambridge, UK) dropwise, then incubated overnight at 4°C. Third, the sections were washed with PBS three times (5 min/time) and incubated with goat anti-rabbit monoclonal antibody at 37°C for 1 hour. Fourth, color development was performed with diaminobenzidine. Each paraffin section was photographed at 6 fields and counted. Also, immunofluorescence technology was made for detecting SMC level.

### RT-qPCR assay

Total RNA was extracted from atherosclerosis samples and control artery samples by the RNAiso Plus (Trizol) kit (Thermofisher, Massachusetts, America), and reverse transcribed to cDNA. RT-qPCR was performed using a Light Cycler® 4800 System with specific primers for TPM2. Table 6 presents the primer sequences used in the experiments. The RQ values (2−ΔΔCt, where Ct is the threshold cycle) of each sample were calculated, and are presented as fold change in gene expression relative to the control group. GAPDH was used as an endogenous control.

**Table 6:** Primers and their sequences for PCR analysis.

**Primer**	**Sequence (5′–3′)**
β-actin-hF	CGCAGAAACGAGACGAGATTG
β-actin-hR	GATGCTCGCTCCAACGACTG
TPM2-hF	TCCACCAAGGAGGACAAATACG
TPM2-hR	GTTGTTGAGTTCCAGCAGGGTC

### Western blot analysis

Abdominal aortic tissues were frozen in liquid nitrogen. Total protein was isolated in a lysis buffer, resolved by 10% sodium dodecyl sulfate polyacrylamide gel electrophoresis (SDS-PAGE) and transferred onto polyvinylidene fluoride (PVDF) membranes by electroblotting. TPM2 protein was detected using an anti-TPM2 polyclonal antibody (1:500 dilution, Bioss, Beijing, China). The bands were visualized with an enhanced chemiluminescence kit (Millipore, Billerica, MA) and analyzed with Image-Pro Plus 6.0 (Media Cybernetics, US).

### The construction of neural network model

The training group was randomly divided into calibration data and training data according to the proportion of 1:2.46. There were 13 samples in the calibration data, and 32 samples in the training data. We used Matlab (version 8.3) to accomplish the normalization processing of variable values, network simulation, network training, and network initialization. The number of input neurons in input layer is same as the number of input variables, and the number is two. The hidden layer is designed as 1 layer, and the output layer is also designed for 1 layer. One output variable is intima-media thickness. When training to 2000 steps after repeated training, the falling gradient is 0, and the training speed is uniform. At the same time, the training error is 0.0064465, and the R (relativity) value reached 0.99133.

### Statistical analysis

The results are presented as the mean ± standard error of the mean. When two groups were compared, an unpaired Student’s t-test was performed to determine statistical significance. The Pearson-rho test was executed to compare the expression of hub genes and status of atherosclerosis for the correlation analysis. When any analytic results reached a liberal statistical threshold of p < 0.2 for each comparison, the risk factors were forced into the univariable linear regression model to confirm independent risk factors for the status of atherosclerosis. Univariate and multivariate logistic regression analysis was used to calculate the odds ratios (ORs) of each hub gene expression for the status of atherosclerosis. The receiver operator characteristic (ROC) curve analysis was performed to determine the usefulness of TPM2 for predicting AS. The Pearson-rho test was executed to compare intima-media thickness, the relative expression of TPM2 and SMC level for the correlation analysis. The cubic spline interpolation algorithm was implemented to analyze the high-risk warning range of atherosclerosis. The statistical analyses were conducted using SPSS software, version 21.0 (IBM Corp., Armonk, NY, USA). P<0.05 was considered to indicate a statistically significant difference.

### Ethics approval

All experiments were approved by Animal Care and Use Committee of the Institute of Laboratory Animal Sciences, Chinese Academy of Medical Sciences (CAMS) & Peking Union Medical College. All institutional and national guidelines for the care and use of laboratory animals were followed.
